# Analysis of functional urological surgery trends 2013–2024 in England using the HES database

**DOI:** 10.1007/s00345-026-06184-9

**Published:** 2026-01-14

**Authors:** Martina Spazzapan, Rhys Evans, Shubhangi Mehta, Sachin Malde, Arun Sahai

**Affiliations:** 1 Department of Urology, Guy’s and St Thomas’ NHS Foundation Trust, London, UK; 2 King’s College London, King’s Clinical Academic Training Office, London, UK; 3 King’s College London, Guy’s, King’s and St. Thomas’ Medical School, London, UK; 4 University College London, London, United Kingdom

**Keywords:** Benign prostate enlargement surgery, Outflow obstruction surgery, Stress incontinence surgery, Urge incontinence surgery, LUTS, Real-world evidence

## Abstract

**Objective:**

To review recent trends in common functional urology surgery in England, using the Hospital Episode Statistics (HES) database.

**Patients and methods:**

Data between 2013 and 2024 were obtained from the HES database, a publicly available dataset recording details about procedures in NHS England. We recorded the procedures for treatment of female stress urinary incontinence (SUI), urgency urinary incontinence (UUI), post-prostatectomy incontinence (PPI), vesicovaginal fistula (VVF) and benign prostatic enlargement (BPE) surgery.

**Results:**

Early in the study period, the most common procedures for SUI were insertion of transobturator and tension-free vaginal tape (8319 procedures/year). We observe an almost complete halt in tape insertion and an increase in removal from 2019 (8 procedures/year), due to concerns about complications and the ‘mesh pause’ in the UK. Injection of bulking agents became the mainstream treatment from 2019 (2490 procedures/year). For refractory UUI, intravesical Botulinum toxin A (BTX) remains the preferred treatment modality (9842 procedures/year). No significant increases in neuromodulation were observed (762/year), and numbers remain low for ileocystoplasty (100/year). Transurethral Resection of the Prostate (TURP) remains the commonest operation for BPE with 15,579 cases in 2023–2024. Laser resection modalities (Holmium laser Enucleation, or HoLEP, and Photoselective Vaporization of the Prostate, or PVP) saw increases with 5098 cases in 2023–2024. VVF repair numbers remain low, averaging 83 per year. There was a reduction in overall surgical numbers during the COVID-19 pandemic, with a partial recovery from 2022 onwards.

**Conclusion:**

Due to concerns around tape-related complications, bulking agents are now the mainstream treatment of SUI in women, and BTX in UUI. Whilst the use of laser is becoming increasingly popular, TURP remains the commonest procedure performed for BPE. There has only been a partial recovery in surgical numbers following the pandemic.

## Introduction

Benign lower urinary tract dysfunction is a major health burden across the globe [1–3].

In men, Benign Prostatic Enlargement (BPE) has an estimated lifetime prevalence of up to 42%, and post prostatectomy incontinence (PPI) affects 2–90% of patients depending on the definition used, with up to 5% requiring surgical management [4, 5]. In women, the UK prevalence of urinary incontinence (UI) is about 40%, with mixed or stress incontinence (SUI) considered the most common [6]. Globally, urgency urinary incontinence (UUI) is estimated to affect up to 36% across both genders [7]. Vesicovaginal fistulas (VVFs) are relatively rare in developed countries like the UK, most commonly occurring as complications following pelvic surgery or radiotherapy. A UK study estimated that VVF complicates approximately 0.12% of hysterectomies [8].

Several techniques have been introduced in the treatment of BPE in recent years, leading to changes in clinical guidelines accounting for prostate size and patient preference [9, 10]. Transurethral resection of the prostate (TURP) remains the recommended standard for prostates between 30 and 80 mL, with bipolar TURP preferred due to a lower risk of TUR syndrome. Photoselective vaporization of the prostate (PVP) is a suitable alternative, particularly for anticoagulated patients. Holmium laser enucleation of the prostate (HoLEP) can be used for prostates of any sizes, and is the recommended treatment for larger prostates (> 80 mL) due to its efficacy and durability. Open simple prostatectomy (OSP) is reserved for very large prostates when endoscopic enucleation is not feasible. Minimally invasive surgical therapies (MIST), such as UroLift and Rezum, are also included in guidelines for patients with prostates < 80 mL, particularly in patients prioritising preservation of ejaculatory function. Urolift and Rezum have gradually been introduced in the UK starting from 2018. Aquablation, suitable for prostates of any size, was introduced at a later stage in selected centres.

Clinical guidelines support the surgical management of UI, encouraging shared decision making. For SUI, options include open, laparoscopic, or robotic colposuspension; autologous “traditional” slings; bulking agents; and synthetic mid-urethral slings via the retropubic or transobturator routes [11, 12]. For UUI, Botulinum toxin-A (BTX) intradetrusor injections and sacral neuromodulation (SNM) can both be offered in refractory cases [12, 13]. Augmentation cystoplasty is considered in severe, refractory cases where bladder capacity is significantly impaired, while urinary diversion (e.g., ileal conduit) is a last resort for patients with end stage bladder dysfunction unresponsive to all other therapies.

For PPI, surgical management depends on severity [14]. The artificial urinary sphincter (AUS) remains the gold standard for moderate-to-severe PPI, offering long-term durability [15]. Male slings are suitable for mild-to-moderate cases, particularly in men with preserved sphincter function [16]. For VVF, surgical repair is typically required, with the approach depending on the location and complexity of the fistula.

The introduction of newer surgical techniques, the Cumberledge report with ‘mesh’ pause, and the COVID-19 pandemic has led to changes in the principles of management of UI and BPE over the last decade [17]. To investigate these changes within the NHS in England, public domain data from the Hospital Episode Statistics (HES) database were interrogated.

## Methods

Public domain data were extracted from the HES database, a curated data product containing details about admissions, outpatient appointments and emergency attendances at NHS hospitals in England. Diagnoses are coded using the International Classification of Diseases and operations are coded using the Office of Population Censuses and Surveys Surgical Operations and Procedures, Fourth Edition (OPCS-4) [18, 19]. We retrieved data on procedures listed in Table 1. Due to OPCS-4 rules, all BPE procedures employing lasers were coded as “Endoscopic Resection of prostate using laser”, including HoLEP, PVP, and enucleation using other lasers (e.g. Thulium laser). Other than the introduction of newer BPE techniques, clinical coding has remained consistent through the last two decades, allowing for longitudinal comparisons [20]. Aquablation and bipolar enucleation were not included as dedicated OPCS 4-character codes for these have not yet been introduced. Similarly, prostate artery embolisation was excluded from this study as the relevant OPCS code (L71.3) encompasses other intraluminal embolisations (e.g. of the uterine arteries for fibroids).

All the data from these codes were merged into summary tables that were then separated into yearly procedures for the period 2013–2024. Information about patient age, median length of stay, and median waiting time was also extracted. A Freedom of Information request was made to NHS England to obtain figures on centres performing VVF repair. Statistical analysis was performed on R, and included Chi Square testing and linear regression models. Our findings are reported according to the Strengthening the Reporting of Observational Studies in Epidemiology (STROBE) guideline [21].

**Table 1:** Surgical procedures for SUI, UUI, PPI, VVF and BPE and their four character codes extracted from the HES database [19]

Indication	Procedure	OPCS code
Female SUI	Fascial sling	M51.1 + M51.8
	Colposuspension	M52.3
	Introduction of TVT	M53.3
	Total removal of TVT	M53.4
	Partial removal of TVT	M53.5
	Introduction of TOT	M53.6
	Total removal of TOT	M53.7
	Bulking agent	M56.3
Urge urinary incontinence	Ileocystoplasty	M36.2
	Botulinum toxin A injection	M49.5 + M43.4
	Sacral neuromodulation	A701
Male SUI/ Post Prostatectomy Incontinence	Implantation of AUS	M64.2
	Male sling	M64.7
Benign prostatic enlargement	Transurethral resection of the prostate	M65.1 + M65.3
	HoLEP and PVP	M65.4
	Rezum	M65.6
	Urolift	M68.3
Vesicovaginal fistula	Vesicovaginal fistula repair	P25.1

*SUI* stress urinary incontinence, *TVT* tension-free vaginal tape, *TOT* transobturator tape, *AUS* artificial urinary sphincter, *HoLEP* holmium laser enucleation of the prostate, *PVP* greenlight laser photovaporisation of the prostate, *OPCS* office of population censuses and surveys surgical operations and procedures

## Results

**Fig. 1:**
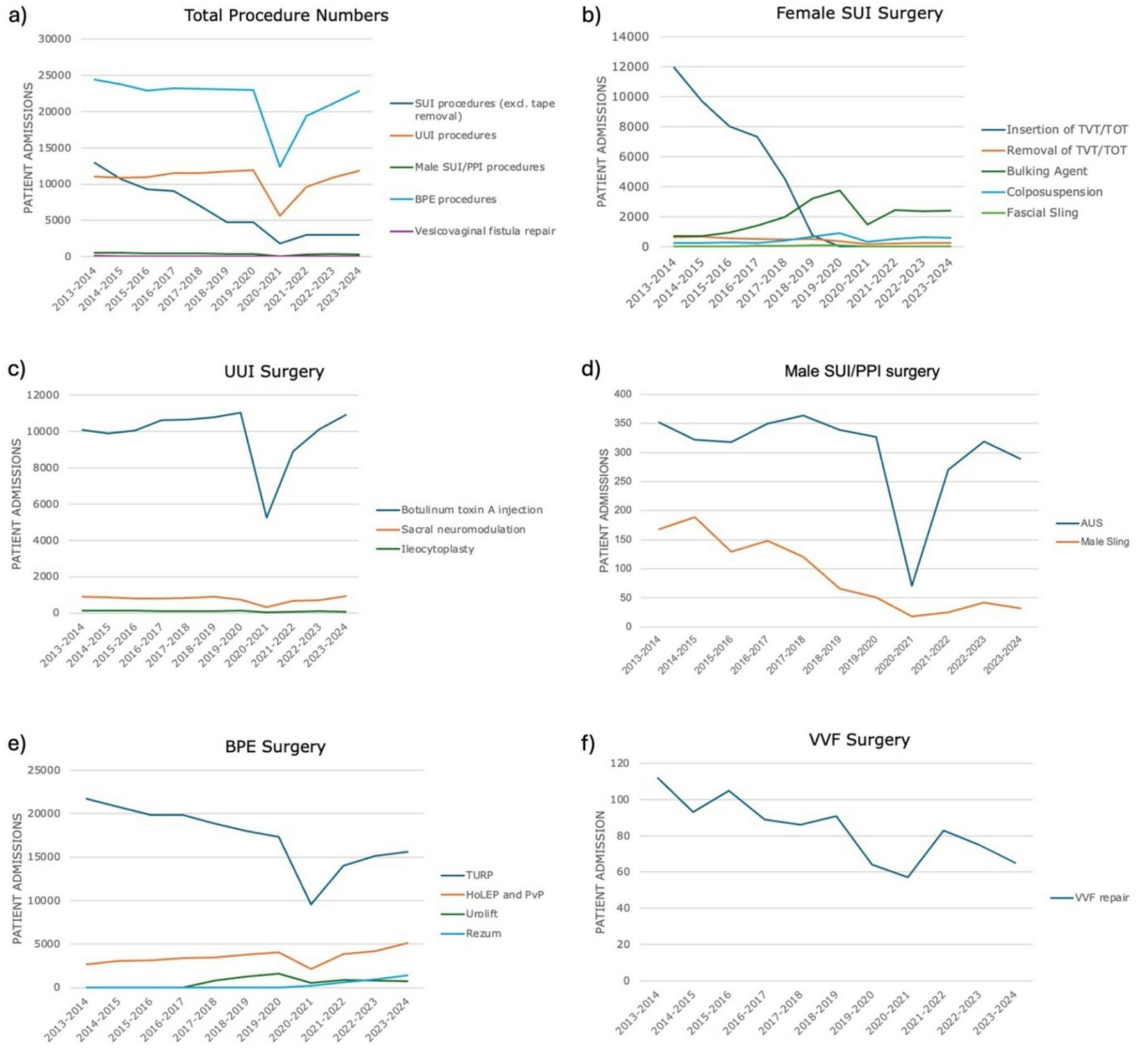
Operative numbers in functional and outflow surgery. **a** operative numbers by category; **b** female SUI surgery; **c** UUI urgery; **d** Male SUI/PPI; **e** BPE surgery; **f** VVF repair

Table 2; Fig. 1 summarise our findings across incontinence, outflow obstruction and VVF surgery. Table 3 summarises median length of stay per procedure, by year.

**Table 2:** Procedures, in numbers of admissions, by year (2013–2024), with linear regression analysis

Procedure	2013–2014	2014–2015	2015–2016	2016–2017	2017–2018	2018–2019	2019–2020	2020–2021	2021–2022	2022–2023	2023–2024	Slope_b1	*p* value	R_squared	CI_Lower	CI_Upper	Significance	Trend
SUI procedures																		
Introduction of TVT	7613	6345	5390	4968	3204	568	14	3	8	5	3	– 842.5	< 0.01	0.861	– 1097.39	– 587.63	***	Decreasing
Introduction of TOT	4353	3396	2650	2360	1316	197	4	2	0	1	1	– 448.3	< 0.01	0.841	– 595.6	– 301.1	***	Decreasing
Partial removal of TVT	348	356	298	256	242	241	149	71	82	98	99	– 30.8	< 0.01	0.899	– 38.58	– 23.02	***	Decreasing
Total removal of TVT	196	197	169	171	165	185	143	80	94	104	69	13.1	< 0.01	0.822	– 17.63	– 8.48	***	Decreasing
Removal of TOT	105	112	109	92	69	88	64	37	53	61	69	– 6.1	< 0.01	0.664	– 9.32	– 2.81	**	Decreasing
Bulking Agent	711	696	939	1402	1994	3207	3769	1479	2432	2368	2405	196.1	< 0.05	0.416	21	371.11	*	Increasing
Colposuspension	248	247	281	256	393	690	898	346	530	649	608	44.0	< 0.05	0.436	6.29	81.71	*	Increasing
Fascial SSling	11	38	30	56	51	80	109	23	21	19	21	– 0.6	0.86	0.004	– 7.4	6.29		Not significant
All SUI (excluding tape removal)	12,936	10,722	9290	9042	6958	4742	4794	1853	2991	3042	3038	– 1051.4	< 0.05	0.886	– 1335	– 767.7	***	Decreasing
UUI procedures																		
Botulinum toxin A injection	10,075	9885	10,056	10,626	10,637	10,789	11,040	5248	8891	10,119	10,901	– 79.84	0.64	0.026	– 447.75	288.08		Not significant
Sacral neuromodulation	881	848	809	805	826	892	745	307	671	692	911	– 17.86	0.29	0.121	– 54.11	18.39		Not significant
Ileocytoplasty	116	131	129	107	101	105	126	45	82	89	71	– 5.75	< 0.05	0.512	– 9.99	– 1.52	*	Decreasing
All UUI	11,072	10,864	10,994	11,538	11,564	11,786	11,911	5600	9644	10,900	11,883	– 103.5	0.58	0.036	– 508.5	301.6		Not significant
Male SUI/PPI procedures																		
AUS	352	322	318	350	364	339	327	71	270	319	289	– 9.69	0.23	0.156	– 26.68	7.29		Not significant
Male Sling	168	189	129	148	120	66	51	18	25	42	32	– 17.35	< 0.01	0.853	– 22.79	– 11.92	***	Decreasing
All male SUI/PPI	520	511	447	498	484	405	378	89	295	361	321	– 27	< 0.05	0.5	– 47.4	– 6.7	*	Decreasing
BPE procedures																		
TURP	21,685	20,770	19,833	19,829	18,879	18,001	17,364	9557	14,037	15,129	15,579	– 841.28	< 0.01	0.621	– 1336.69	– 345.87	**	Decreasing
HoLEP and PvP	2694	3034	3095	3387	3482	3760	4035	2113	3884	4209	5098	155.38	< 0.05	0.407	14.1	296.67	*	Increasing
Urolift	0	0	0	0	779	1295	1597	535	898	791	710	102.69	< 0.05	0.375	2.79	202.59	*	Increasing
Rezum	0	0	0	0	0	0	0	207	595	930	1415	118.13	< 0.01	0.649	52.68	183.58	**	Increasing
All BPE	24,379	23,804	22,928	23,216	23,140	23,056	22,996	12,412	19,414	21,059	22,802	– 465.1	0.16	0.209	– 1148.3	218.1		Not significant
VVF repair																		
All VVF	112	93	105	89	86	91	64	57	83	75	65	– 4.17	< 0.01	0.648	– 6.49	– 1.85	**	Decreasing
Centres performing 10 or more repair a year	2	2	1	1	2	2	0	1	2	2	2							

**Table 3:** Median length of stay per procedure, per year (2013–2024), with linear regression analysis

Procedure	2013–2014	2014–2015	2015–2016	2016–2017	2017–2018	2018–2019	2019–2020	2020–2021	2021–2022	2022–2023	2023–2024	Slope_b1	*p* value	R_squared	CI_Lower	CI_Upper	Significance	Trend
SUI procedures																		
Introduction of TVT	1	1	1	1	1	1	1	2	2	2	2	0.1273	< 0.01	0.7	0.0644	0.1901	**	Increasing
Introduction of TOT	1	1	1	1	1	1	3	1	N/A	3	3	0.2178	< 0.05	0.565	0.062	0.3735	*	Increasing
Partial removal of TVT	1	1	1	1	1	1	1	1	1	2	3	0.1273	< 0.05	0.426	0.0159	0.2387	*	Increasing
Total removal of TVT	1	2	1	1	2	1	2	3	3	3	4	0.2636	< 0.01	0.701	0.1338	0.3935	**	Increasing
Removal of TOT	1	1	1	1	2	2	2	3	3	3	3	0.2545	< 0.01	0.891	0.1874	0.3217	***	Increasing
Bulking Agent	1	1	1	1	1	1	1	1	1	1	1	0	0.12	0.527	0	0		Not significant
Colposuspension	3	3	3	3	2	2	2	2	2	2	2	– 0.1273	< 0.01	0.7	– 0.1901	– 0.0644	**	Decreasing
Fascial Sling	2	2	2	1	1	2	2	2	2	2	2	0.0273	0.51	0.05	– 0.0624	0.1169		Not significant
UUI procedures																		
Botulinum toxin A injection	1	1	1	1	1	1	1	1	1	1	1	0	0.12	0.527	0	0		Not significant
Sacral neuromodulation	1	1	1	1	1	1	1	1	1	1	1	0	0.12	0.527	0	0		Not significant
Ileocytoplasty	9	10	10	9	9	9	10	8	9	10	10	0.0091	0.90	0.002	– 0.144	0.1622		Not significant
Male SUI/PPI procedures																		
AUS	2	1	1	1	1	1	1	1	1	1	1	– 0.0455	0.12	0.25	– 0.1048	0.0139		Not significant
Male Sling	1	1	1	1	1	1	1	1	1	1	1	0	0.12	0.527	0	0		Not significant
BPE procedures																		
TURP	2	2	2	2	2	2	2	2	2	2	1	– 0.0455	0.12	0.25	– 0.1048	0.0139		Not significant
HoLEP and PvP	1	1	1	1	1	1	1	1	1	1	1	0	0.12	0.527	0	0		Not significant
Urolift	N/A	N/A	N/A	N/A	1	1	1	1	1	1	1	0	0.14	0	0	0		Not significant
Rezum	N/A	N/A	N/A	N/A	N/A	N/A	N/A	1	1	1	1	0	N/A	N/A	0	0		Insufficient data
VVF repair																		
Vesicovaginal fistula repair	6	6	5	5	5	4	4	3	4	4	4	– 0.2364	< 0.01	0.704	– 0.3519	– 0.1208	**	Decreasing

### Female SUI surgery

Drastic changes are observed in the study decade with an overall significant downtrend in surgical numbers. Initially the most common procedure was tape insertion (transvaginal tape, or TVT, and transobturator tape, or TOT), averaging 8319 procedures per year in the period 2013–2018 (SD 2775). We observe an almost complete halt in tape insertion from 2019 onwards, with an average of 8 insertion/year (SD 5); With regards to tape removal, there were on average 425 procedures per year across the study decade (SD176). Downtrends were significant for both tape insertion and removal.

The use of bulking agents showed a significant uptrend, surpassing tape insertion in 2019 and becoming the mainstay treatment for SUI (2490 procedures/year in the period 2019–2024, SD 819). Colposuspension numbers showed a small but significant increase across the study decade, with an average of 467 procedures per year (SD 221). Fascial sling insertion saw a brief increase in numbers across 2018–2020 which was not stastistically significant nor sustained, with an average of 21 procedures per year (SD 1.6) from 2021 onwards.

The mean age of patients undergoing tape insertion and removal was 52.3 years (SD 14.7) and 56 years (SD 15.6) respectively. The mean age for female sling insertion and urethral bulking was 53 years (SD 14.7) and 55.9 years (SD 17.8) respectively.

With regards to length of stay, both tape insertion and removal saw significant increases over the study decade. There were no changes for bulking and fascial sling insertion, while we found a significant decrease in length of stay for colposuspension.

**Fig. 2:**
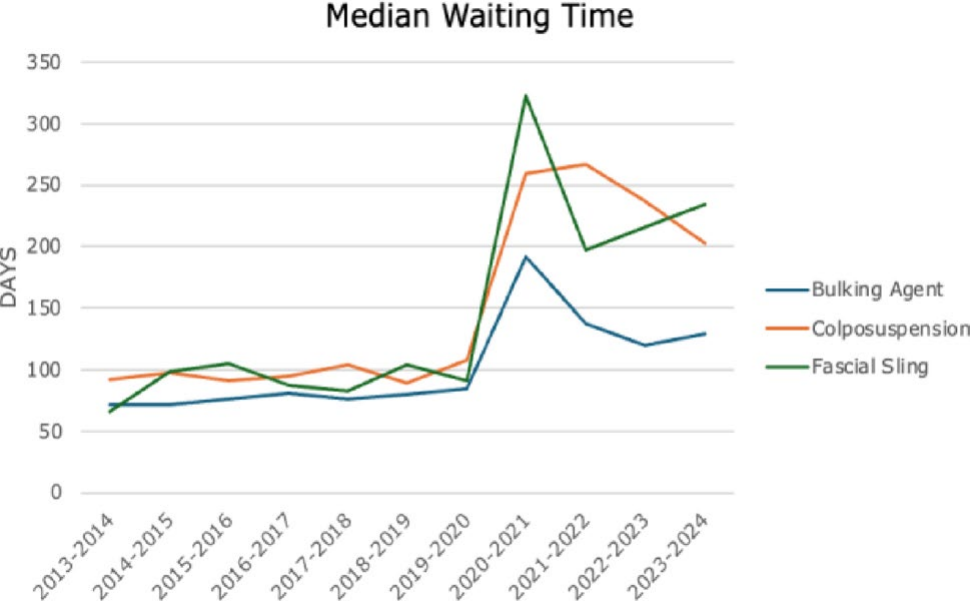
Median waiting time in days for non-mesh SUI procedures

Median waiting time for non-mesh SUI procedures is displayed in Fig. 2. Overall median time was 88 days (SD 12) for colposuspension, bulking, and sling insertion until 2019–2020, and increased to 210 (SD 60) from 2020 to 2021 onwards (*p* < 0.01).

### UUI surgery

There were no significant changes in overall UUI numbers. Intravesical BTX remained the treatment modality of choice across the study decade, despite a temporary reduction in numbers in 2019–2021, corresponding to the COVID-19 pandemic. Across the study period, there were an average of 9842 procedures/year (SD 1639) with no significant downtrend. Lower numbers are seen for neuromodulation (average 762/year, SD 170) with no significant trend observed. Numbers are even lower in ileocystoplasty (average 100/year, SD SD 26), with a significant downtrend.

Age distribution between treatment modalities for refractory UUI varied: the mean age for patients undergoing BTX and SNM was 55.1 years (SD 18.9) and 50.4 years (SD 15.5) respectively. Ileocystoplasty patients were younger with mean age of 26.6 years (SD18.9, *p* < 0.01). The mean age of patient undergoing ileocystoplasty fell from 34 years old (SD 17.3) in the decade 2003–2013 to 26.6 (SD 18.9) in our cohort (*p* < 0.01) [20]. No significant trends were observed across procedures with regards to length of stay.

### Male SUI/PPI surgery

There was an overall significant decrease in male SUI surgery across the study decade. AUS remained the mainstay approach for male SUI/PPI (average 302 procedures/year, SD 81), with no significant trend observed. However, there was a significant downtrend in use of male sling (average 90 procedures per year, SD 62). Again, a reduction in numbers in the period 2019–2021 is noted, in keeping with reduced benign operative numbers during the pandemic.

The mean age of patients undergoing AUS and sling insertion was 66.6 years (SD 13.1) and 67.1 (14.0) years respectively. No significant trends were observed across procedures with regards to length of stay.

### BPE surgery

Overall BPE surgical numbers remained static through the study decade despite a temporary reduction in cases during the pandemic period. TURP remains the mainstay operation for BPE (average 17333/year, SD 3540), though a significant downtrend is observed. This was observed in conjunction with a significant increase in other resection modalities, with laser procedures (primarily HoLEP and PVP) representing the second most common choice (average 3426/year, SD 807), followed by minimally invasive techniques (Urolift average 943/year, SD 370; Rezum average 786/year, SD 512). Rezum was first introduced in 2020, and from 2022 we observe higher numbers than Urolift, despite its earlier introduction. An overall positive trend was observed for both Urolift and Rezum.

The mean ages for patients undergoing BPE surgery were 72.3 years (SD11.5) for TURP, 71.7 years (SD 11.4) for laser procedures, and 68 years for both Urolift (SD14.9) and Rezum (SD 13.7). No significant trends were observed across procedures with regards to length of stay.

### VVF repair

There was a significant downtrend in the procedures carried out, with 112 procedures carried out in 2013–2014, and 65 in 2023–2024 (average across the study period 84 procedures/year, SD 17.2). VVF repair was performed in 114 different centres in England across the study decade in its entirety. However, on any one year, only one or two centres performed 10 or more yearly repairs. The average age for VVF repair was 52.1 years (SD 17.2). There was an overall significant decrease in length of stay across the study decade.

## Discussion

This study provides a comprehensive overview of the evolving landscape of UI, BPE and VVF procedures in England over the past decade, highlighting significant shifts in clinical practice within the decade, and in comparison to the period 2003–2013 [20].

### SUI surgery

The marked decline in the use of synthetic mid-urethral tapes for SUI, initiated around 2019, aligns with the findings of the Cumberlege Review, which highlighted concerns regarding mesh-related complications and formalised pathways for tape removal procedures, managed by nine specialist centres across England [17]. The increased length of stay for mesh-related procedures across the decade likely reflects an increase in surgical complexity. Reduced length of stay was observed in colposuspension, likely as a result of the introduction of minimally invasive approaches for the procedure.

The “mesh pause” created a treatment gap that has been predominantly filled by the adoption of bulking agents. These agents offer advantages such as a favourable safety profile and a relatively short learning curve for practitioners [22]. In contrast, alternative options like colposuspension and autologous slings, despite their improved efficacy in relation to bulking agents, have seen limited uptake, possibly due to their increased invasiveness, technical complexity and steeper learning curves [23]. Additionally, UI surgery is performed by both gynaecologists and urologists. The authors hypothesise that urologists are more likely to perform autologous fascial slings than gynaecologists in England. Gynaecologists perform five times as many more SUI procedures compared to urologists, which could partly reflect the lower numbers of fascial slings compared to other SUI surgeries [24].

While the decline in tape insertion following the mesh pause is well documented, the reduction in overall numbers raises important questions about the surgical management of SUI. The increase in bulking agents only partially compensates for the overall sharp drop in numbers, suggesting a potentially significant cohort of patients with untreated or undertreated SUI.

Several hypotheses may explain this gap. First, heightened media coverage and medico-legal concerns may have contributed to patient reluctance or clinician hesitancy, effectively discouraging surgical referrals. Second, the current specialist-led model for managing mesh-related complications might have redirected healthcare resources and clinical attention away from new surgical candidates. Our finding of increased median waiting time across the decade may support this hypothesis.

Third, the authors hypothesise that women were previously offered SUI surgery more readily, but with improved counselling in the aftermath of the mesh pause, some are now managed conservatively or lost to follow-up.

This is an important area for further research, as the reduction in procedural rates could signify a treatment shortfall for a condition that significantly affects quality of life. Understanding the downstream impact on health outcomes, patient satisfaction, and long-term continence management will be critical to shaping future policy and service provision in the post-mesh era.

### UUI surgery

With regards to the management of refractory UUI, BTX injections have maintained their position as the primary treatment modality. This preference is attributed to its cost-effectiveness, ease of administration (i.e., local anaesthesia in outpatient setting), and patient tolerability. Despite clinical guidelines advocating the use of SNM following the failure of medical management, its adoption remains limited. Currently, SNM is available in only 37 centers across England, suggesting potential issues related to accessibility and resource allocation [25]. NHS commissioning for SNM has been complicated in some regions and not uniform throughout England. However, our data show an improvement compared to the previous decade, with 911 cases carried out in 2023–2024 compared to 249 in 2011–2012 and 23 in 2001–2002 [20]. Technology in this field is evolving and several companies are now in the market with their own devices. Increased competition has led to smaller devices, MRI compatibility, rechargeable and more durable battery systems which may improve uptake in coming years [26].

Ileocystoplasty numbers remained low, suggesting its ongoing but limited role in refractory overactive bladder / UUI, neurogenic bladder, and severe end stage bladder dysfunction. The low patient age reflects the continued role of augmentation for younger patients affected by neurological disorders and congenital bladder abnormalities.

### Male SUI/PPI surgery

The overall number of interventions for male SUI/PPI showed a decline across the study decade but is anticipated to rise in conjunction with improvements in prostate cancer survivorship care. The AUS continues to be the most common treatment and is the preferred choice for moderate to severe PPI in EAU and AUA guidelines, with male slings presenting as an option for mild to moderate stress urinary incontinence. The reasons behind low sling adoption remain unknown; the authors hypothesise that concerns around female synthetic meshes dampened the adoption of the male sling. It is worth noting that the period 2021–2022 saw an increase in sling numbers, following almost a decade of declining cases. The MASTER trial, a UK-based randomised controlled trial demonstrating sling non-inferiority compared to AUS, was published in early 2021 and may have contributed to increased uptake among UK surgeons [27].

### BPE surgery

The relative stability in overall BPE numbers suggests sustained demand and service provision. However, our data suggest gradual shift away from TURP toward laser procedures and MISTs. This decline in TURP is consistent with global trends, particularly for patients with small-to-medium-sized prostates, those wishing to preserve ejaculatory function, or those deemed unfit for more invasive surgery [28, 29].

Our data did not differentiate between HoLEP and PVP numbers due to laser procedures sharing the same OPCS code. However, recent literature suggests over two thirds of BPE laser procedures are HoLEP, despite its longer learning curve [30]. Thanks to its efficacy in large gland burdens, it is hypothesised that increases in HoLEP numbers mirror a decrease in benign prostatectomy. However, prostatectomy data were not included in this study due to overlap in benign and radical prostatectomy coding. Further disaggregation of procedural codes would be helpful to clarify procedural indications and trends, particularly for distinguishing between energy modalities and enucleation techniques.

Several MISTs are now offered in the NHS for smaller prostates; UroLift and Rezum have been introduced in 2017 and 2020 respectively, with Rezum demonstrating a more rapid uptake. The rising uptake of MISTs reflects a possible re-orientation toward earlier surgical intervention, both in younger patients and in those previously managed medically or not offered surgery due to frailty or risk.

Other MISTs under investigations include iTIND and Aquablation. iTIND, both of which have been approved by the National Institute for Health and Care Excellence. Aquablation is available for the treatment of prostates of any size, however data on its introduction in England were not available at the time of writing. It is hypothesised that its shorter learning curve compared to HoLEP may facilitate quicker integration into clinical practice for prostates > 100 cc. Lastly, Prostate Artery Embolisation (PAE) is a non-surgical option for BPE currently offered in several NHS centres. Procedure numbers were not available via HES for the purposes of this study as PAE is coded as non-specified endovascular procedure.

The expansion of MISTs raises important questions about its impact on medical management of BPE. Longitudinal studies in the UK and Norway indicate an overall increase in the use of pharmacotherapy for BPE over the last two decades [31, 32]. While our dataset did not include medication data, linkage between HES and prescribing data could investigate whether earlier use of MIST is mirrored by a reduction in long-term pharmacotherapy or delayed progression to more invasive surgery.

### VVF repair

The incidence of VVF surgeries is low, ranging 64–112 cases per year over the decade (excluding the first year of the COVID pandemic) with a significant downtrend. 114 centres performed VVF repairs over study period as a whole, but only two institutions handled over ten cases during any year. This distribution raises considerations for centralising care to specialised centers to ensure procedural proficiency and enhanced surgical outcomes. Geography and access are also of importance when considering how many centers would be appropriate for this type of surgery. There was a significant reduction in length of stay across the study decade, likely reflecting standardised postoperative protocols such as the Enhanced Recovery after Surgery approach.

### General trends and limitations

A significant decline in the volume of all aforementioned surgeries was observed during the COVID-19 pandemic, with only a partial recovery noted in subsequent years. This trend underscores the pandemic’s enduring impact on elective surgical services and highlights the need for strategic planning to address the backlog and meet ongoing patient care demands.

This study involved use of the HES database, which provides large-scale, centralised coding data from NHS England. While there is variation in the quality of clinical coding, HES coding is considered robust for research purposes [33]. With regards to outflow obstruction surgery, there has been emphasis on improving coding accuracy leading to guidance from the Getting It Right First Time coding team [34]. Nevertheless, the OPCS coding structure used by HES has significant limitations, such as the inability to distinguish between different laser modalities for BPE surgery, or lack of specific codes for pulmonary artery embolisation and aquablation.

Reliance on the HES database also limits opportunities for analysis of patient preferences, postoperative outcomes and complications. Additionally, the study includes information about procedures carried out in England alone, rather than the whole United Kingdom, as it relies on the HES database which is curated by NHS England. Further, the study cannot account for regional variability or assess disparities in access to newer or specialised treatments.

Finally, structural factors such as population ageing, prioritisation of oncological procedures and variation in local surgical capacity all influence procedural numbers. Delays in access, especially following the pandemic, may disproportionately affect older or comorbid patients, introducing bias into the study dataset.

## Conclusion

This study highlights significant shifts in surgical practices for UI and BPE in England, influenced by evolving clinical guidelines, emerging technologies, and external factors such as the COVID-19 pandemic. Evolving concerns around tape-related complications have led to a shift toward bulking agents as the predominant treatment for female SUI, while BTX remains central in the management of UUI. Despite the growing adoption of HoLEP and PVP, TURP continues to be the primary surgical approach for BPE. Ongoing surveillance of these trends is essential to inform healthcare planning and resource allocation, ensuring optimal patient outcomes.

## Data Availability

No datasets were generated or analysed during the current study.
